# A gene-based approach for testing association of rare alleles

**DOI:** 10.1186/1753-6561-5-S9-S7

**Published:** 2011-11-29

**Authors:** Hongyan Xu, Varghese George

**Affiliations:** 1 Department of Biostatistics, Georgia Health Sciences University, 1120 15th Street, Augusta, GA 30912, USA

## Abstract

Rare genetic variants have been shown to be important to the susceptibility of common human diseases. Methods for detecting association of rare genetic variants are drawing much attention. In this report, we applied a gene-based approach to the 200 simulated data sets of unrelated individuals. The test can detect the association of some genes with multiple rare variants.

## Background

Genome-wide association studies (GWAS) have been promising for identifying the underlying genetic basis of complex disorders. Indeed, many disease susceptibility regions have been identified using this approach. However, there is still “missing heritability” for most common diseases [1]. Part of the reason is that the statistical tests used in traditional GWAS may not have sufficient power to detect the association of rare genetic variants because of low allele counts in a sample. Rare genetic variants have been shown to contribute to the risk in some common disorders [2,3]. A possible approach is to combine the information at multiple rare genetic variants and test the association collectively in a gene or a pathway [4,5]. Dering et al. [6] provides a review of the association methods that combine information from multiple genetic markers. Here, we apply a gene-based approach for testing association of rare alleles [7] to the 200 simulated data sets of unrelated individuals provided by Genetic Analysis Workshop 17. The empirical type I error rate and power are reported.

## Methods

### Statistical test

We apply an association method to combine the information of single-nucleotide polymorphisms (SNPs) in a particular gene. The method can be applied to any segment of the genome. Here, we use genes as natural segments of the genome. Instead of using the standard chi-square test to compare the allele frequencies in case and control subjects, we propose to compare the mutation rates between the two groups. Specifically, we count the number of minor alleles with a minor allele frequency (MAF) less than 0.01 (which we refer to as mutations) in a specific gene in each individual from the case and control groups. Let *P_i_* be the number of mutations in a gene in individual *i* in the case group and *Q_j_* be the number of mutations in the same gene in individual *j* in the control group. Let  and  be the average number of mutations in the gene in the case and control groups, respectively, and let  be the average number of mutations in the gene in the total sample. Also, let  be the pooled sample variance of the number of mutations in the total sample, given by:(1)

where *n_P_* and *n_Q_* are the number of individuals in the case and control groups, respectively, and *N* is the total sample size. Then we define the test statistic:(2)

Under the null hypothesis of no association of the set of SNPs in a gene with the disease, the average number of mutations in the case and control groups should be equal and the test statistic *T_G_* is asymptotically distributed as a central chi-square distribution with one degree of freedom.

## Results

### Distribution of SNPs within gene

We identified all the SNPs within a specific gene by comparing the nucleotide position information against the starting and ending positions of the gene. Gene *AHNAK*, which has 231 SNPs, is the gene with the most SNPs. Nonetheless, 1,191 genes have only one SNP. The mean number of SNPs is 8.33 per gene with a variance of 227.6.

### Type I error

The affection status provided in each phenotype replicate file was used to put the samples into case and control groups. We then applied the gene-based test to all the 200 replicates of the simulated data. As an example, Table 1 lists the genes that are significant at the 0.001 level from the tests using the data from the first replicate.

**Table 1:** Significant genes at the 10^–4^ level from replicate 1

Gene	Chromosome	Gene length (bp)	Number of SNPs	*p*-value
*ADAM15*	1	11,491	30	1.74 × 10^–6^
*FLT1*	13	192,877	35	2.23 × 10^–6^
*RIPK3*	14	4,016	21	8.15 × 10^–6^
*LOC100130230*	5	5,347	10	1.18 × 10^–5^
*PIK3C2B*	1	67,714	71	1.51 × 10^–5^
*MAP3K12*	12	18,992	17	2.73 × 10^–5^
*UAP1*	1	38,338	13	2.82 × 10^–5^
*BCHE*	3	64,562	29	3.26 × 10^–5^
*SUSD2*	22	7,631	45	4.93 × 10^–5^
*OR10H4*	19	951	20	6.50 × 10^–5^
*CD79B*	17	3,607	10	8.57 × 10^–5^

**Figure 1:**
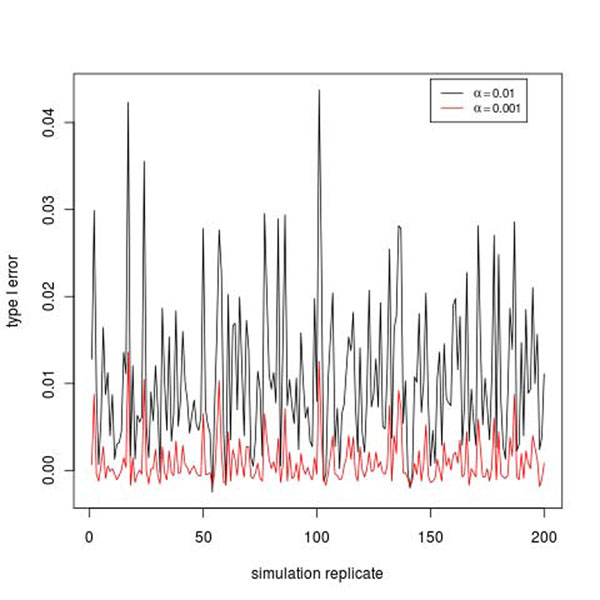
Type I error rate in 200 simulation replicates.

From the simulation model, 36 genes are involved in the simulation of the disease affection status, as explained in the next subsection. Therefore the remaining 3,169 genes are not involved. The significant genes among these 3,169 genes are counted as false positives. For each replicate, we count the number of false positives and calculate the ratio of the number over 3,169. We take the ratio as an estimate of type I error rate. Figure 1 gives the plots of the type I error rate in 200 replicates at the 0.01 and 0.001 levels. The mean and standard deviation of the type I error rate are 0.00105 and 0.00837 at the 0.01 level and 0.00102 and 0.00268 at the 0.001 level.

### Power

**Figure 2:**
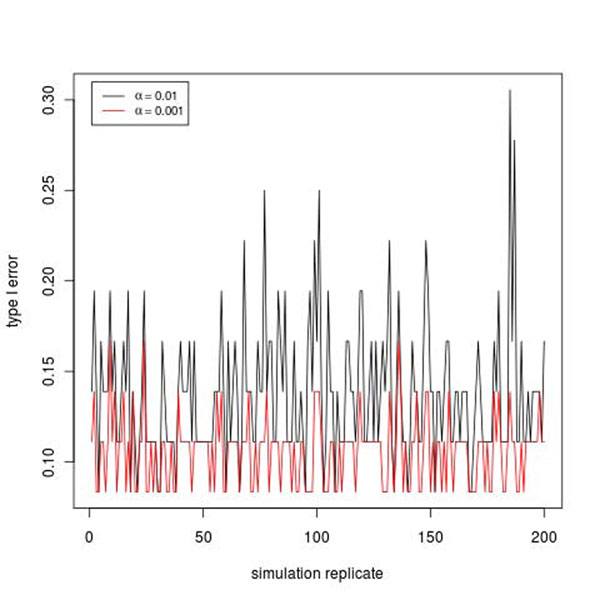
Empirical power in 200 simulation replicates.

Using the simulation model [8], we simulate the disease status using a liability threshold model. The liability is a function of Q1, Q2, Q4, and a latent liability, which are influenced by 36 genes. For the test results of each replicate, we count the number of significant genes that are among the 36 genes, that is, real positives, and calculate the ratio of the number over 36 as a rough estimate of power for each replicate. Six of the 36 genes are significant at the 0.001 level from replicate 1. This gives a power estimate of 16.7%. Figure 2 gives the plot of the power estimates in 200 replicates at the 0.01 and 0.001 levels. The power estimates vary quite a bit across replicates. The mean and standard deviation of the power estimates are 0.137 and 0.037 at the 0.01 level and 0.106 and 0.019 at the 0.001 level.

Because we have test results from 200 replicates, we also count the number of times a particular gene is called significant across the 200 replicates. Table 2 gives the list of genes that are significant at least 30 times over the 200 replicates at the 0.001 level. At the top of the list is the *FLT1* gene, which is significant 171 times at the 0.01 level and 136 times at the 0.001 level, giving power estimates of 85.5% and 68.0%, respectively. From the simulation model, 11 of the 35 SNPs in this gene are influencing Q1. Similarly, the tests at the *PIK3C2B* gene are significant 139 times at the 0.01 level and 87 times at the 0.001 level, giving power estimates of 69.5% and 43.5%, respectively. *PIK3C2B* has 71 SNPs, 24 of which influence the disease liability.

**Table 2:** Number of significant tests across 200 replicates

Gene	Number of significant tests		Chromosome	Gene length (bp)	Number of SNPs
				
	α = 0.01	α = 0.001			
*FLT1*	171	136	13	192,877	35
*PIK3C2B*	139	87	1	67,714	71
*HDAC4*	126	79	2	352,780	17
*NFKBIZ*	132	66	3	33,010	26
*SLC6A3*	116	61	5	52,630	25
*RNF145*	126	52	5	50,416	18
*BRWD2*	51	42	10	58,342	2
*SHC3*	92	36	9	53,358	5
*WNT16*	71	36	7	51,384	20
*TTLL4*	81	33	2	9,654	50

## Discussion

Association methods for rare genetic variants are attracting much attention in the genome era, especially with the advance of next-generation sequencing technology. Because rare alleles appear in only a few individuals, the traditional single-marker tests have low power. An alternative method is to group genetic variants by gene or pathway and test the variants in one group collectively. In this report, we applied a gene-based approach to the data on unrelated individuals. The test is based on the Poisson mutation process for rare genetic variants. Our results show that this test has modest power in detecting the association of genes when all the underlying genes are considered. The type I error rate seems to be well controlled on average but is inflated in some replicates, as shown in Figure 2. However, it should be noted that the approach for estimating the type I error rate is not rigorous in that in each replicate the estimate is based on only 3,169 “null genes” assumed to be unrelated to the disease status. There could be considerable Monte Carlo error; the assumption that all of the 3,169 genes are unrelated to the disease status may not be true if there are some unknown interactions between some genes used in simulating the disease phenotype and some null genes. It is encouraging to see that the test can detect the association signal at *FLT1* and *PIK3C2B* with relatively good power. Nonetheless, the validity and power of the test depend on the assumption of the distribution of the susceptibility mutations. Apparently, if a particular gene has many susceptibility mutations, then, because all of them are contributing to the disease risk, we would expect a larger difference between the number of mutations in the case and control groups, which could translate into higher power than genes with fewer mutations.

The validity of using the simulated data sets also depends on the simulation model and its compatibility with the test assumptions. Because this is a group test of all the SNPs within one gene, the model might not work well for genes that have only one or two susceptibility mutations, whereas it does work well for genes with more susceptibility mutations, as in the cases of *FLT1* and *PIK3C2B*, which have 11 and 24 susceptibility SNPs, respectively. The simulation model also assumes that all the minor alleles in the model increase disease risk, which may favor some of the collapsing methods.

## Conclusions

The proposed gene-based association method can detect the association of some genes with multiple rare variants.

## Competing interests

The authors declare that there are no competing interests.

## Authors’ contributions

VG participated in the design of the study and interpretation of the statistical analysis. HX conceived of the study and participated in its design and coordination and drafted the manuscript. All authors read and approved the final manuscript.
